# Between Alarms and Scheduling: The Effect of Cognitive Offloading on Prospective and Retrospective Memory

**DOI:** 10.3390/bs16060872

**Published:** 2026-05-31

**Authors:** Joana B. Silva, Pedro B. Albuquerque, Inês B. Oliveira, Pedro F. S. Rodrigues

**Affiliations:** 1 School of Psychology, University of Minho, 4710-057 Braga, Portugal; joanabsilva.151@gmail.com; 2 Department of Psychology and Education, Portucalense University, 4200-072 Porto, Portugal; inesoliveiraify@gmail.com (I.B.O.); prodrigues@upt.pt (P.F.S.R.); 3 RISE-Health (RISE-Health@UPT), Portucalense University, 4200-072 Porto, Portugal

**Keywords:** cognitive offloading, prospective memory, retrospective memory, metamemory, rehearsal strategies

## Abstract

Prospective memory (i.e., remembering to complete future plans) and retrospective memory (i.e., memory for past events) are essential to daily functioning, but both are prone to everyday failures, such as forgetting to carry out an intended action (e.g., missing a medication dose) or inaccurately recalling past information (e.g., forgetting the details of a recent conversation). To mitigate these, individuals may rely on cognitive offloading, the use of physical actions or external tools to reduce retrieval effort (e.g., setting an alarm to avoid forgetting an important task). This study examined the impact of cognitive offloading on both prospective and retrospective memory, using two offloading strategies. In the first phase, 152 participants were instructed to send an email 48 h later at 7 p.m. and were randomly assigned to one of three conditions: a reminder (e.g., an alarm), a scheduled email for automatic delivery, or no reminder (internal memory). They also watched a news report. In the second phase, participants sent an email (prospective memory) and then completed a free recall question about the video (retrospective memory). Results show that both offloading conditions performed better in the prospective task. Notably, there were no significant differences in retrospective memory performance. Overall, cognitive offloading enhanced prospective memory and subjective confidence but did not influence retrospective recall, highlighting a dissociation between remembering when to act and remembering contextual information.

## 1. Introduction

Remembering future intentions and recalling past events are essential for effective everyday functioning (Talbot et al., 2018). Despite extensive research on how individuals form and retrieve intentions, there remains a limited understanding of how contemporary cognitive strategies, particularly those involving technological or environmental aids, influence these memory processes. This question is increasingly relevant in daily life, where reliance on digital reminders and external supports is pervasive and may fundamentally alter the way intentions are encoded and retrieved. Prospective memory refers to the capacity to create, store, and perform an intention at a specific time in the future in response to an exact cue (Zogg et al., 2012). It describes a person’s ability to recall a specific action at a particular moment (Einstein et al., 1998; Talbot et al., 2018). It consists of two components: (1) a prospective component; (2) a retrospective component (Einstein & McDaniel, 1996). Prospective memory involves remembering something we need to do in the future, along with the specific time and date it needs to be done (e.g., remembering to take our medication at a particular time and date). Retrospective memory involves recalling an event or information about something that has already occurred, such as remembering how to take medication (Richmond et al., 2025; Talbot et al., 2018).

Both prospective and retrospective components are theorized to be singly dissociable, as retrospective memory is a necessary but insufficient condition for successful prospective memory (Zogg et al., 2012). Prospective memory, on the other hand, may be shaped by multiple factors, such as the context in which it is planned, recalled, and should take place, working memory capacity, social and motivational aspects, and the memory strategies implemented (Ellis & Kvavilashvili, 2000; Richmond et al., 2025). From a metacognitive perspective, these strategies can be viewed as compensatory mechanisms: individuals evaluate their cognitive resources and decide whether to rely on internal rehearsal or delegate the task to external aids. Understanding how such metacognitive judgments influence the adoption and effectiveness of offloading strategies is, therefore, central to explaining prospective memory performance.

The literature features a widely accepted conceptual model of prospective memory (McDaniel & Einstein, 2000; Zogg et al., 2012) that describes a multi-stage process. The first stage involves forming an intention with an associated cue, which may be based on the passage of time or a specific event. The second stage consists of a delay between the formation of the intention and its execution, which can range from minutes to weeks. The third stage involves accurately detecting and recognizing the cue, resulting in a self-initiated retrieval of the associated intention. Several factors can influence detection and recognition at this stage, including the cue’s salience, its relevance to the ongoing task, its association with the intention, and whether it requires detecting the passage of time or the occurrence of an event. Time-based cues are more challenging to detect as they place greater demands on strategic monitoring, whereas event-based cues tend to involve more automatic or spontaneous retrieval processes. The final stages include recalling and executing the intention. Retrospective memory is responsible for recalling the content of the intention, and its accuracy may be influenced by the complexity of the information, including the volume, relevance to the intention, and the number of competing intentions.

As mentioned, both prospective and retrospective memories are fundamental in daily tasks. However, lapses do occur, leading to failures that may pose significant risks to an individual’s health and safety, given that these cognitive processes are vital for sustaining independence, employment, and social interactions (Foster et al., 2009). To minimize these lapses, individuals can implement various cognitive strategies. People who are highly confident in their memory and exhibit higher metacognition are less likely to use such cognitive strategies, whereas people who are less confident in their memory rely on them more often (Fellers et al., 2023; Risko & Gilbert, 2016). Some common cognitive strategies include rehearsals or cognitive offloading.

On the one hand, rehearsals can be triggered by external cues (e.g., smelling coffee and remembering to buy it at the supermarket) or internal cues (e.g., making a mental to-do list and remembering a pending task). This happens in between forming and executing an intention (Fellers et al., 2023). A higher frequency of rehearsals has been associated with improved prospective memory performance (Kvavilashvili & Fisher, 2007).

On the other hand, cognitive offloading refers to the strategic use of physical actions or external tools to facilitate information processing and reduce cognitive load. A typical example is writing down a friend’s birthday on your planner as an external aid, rather than relying solely on memory. Additionally, cognitive offloading helps us overcome cognitive limitations by manipulating our surroundings or bodily actions (e.g., turning one’s head to view an upside-down image rather than mentally rotating it) (Risko et al., 2014; Risko & Gilbert, 2016). Risko and Gilbert (2016) identified several forms of cognitive offloading. One form is external normalization, in which individuals manipulate the environment to support cognitive tasks (e.g., using fingers to count for simple addition). Another is intention offloading, which involves external reminders to track future tasks (e.g., writing reminders on calendars or using Post-it notes).

In everyday life, individuals frequently implement cognitive offloading strategies, often without consciously planning them. One example is the widespread habit of taking numerous pictures of various events (e.g., experiences, objects, people) to preserve memories. However, sometimes, implementing cognitive offloading strategies may not work as intended. In some cases, implementing these strategies can be less effective than relying solely on internal memory for enhancing memory performance. Soares and Storm (2018) found that people remember observed objects better than those they photographed when they no longer had access to the photographs. However, current findings remain mixed regarding whether offloading consistently benefits memory performance. Some studies have shown that relying on external tools can improve task accuracy by reducing cognitive demands (e.g., Burnett & Richmond, 2023), whereas others report negative consequences when those tools are no longer available (Henkel, 2014; Soares & Storm, 2022). This ambiguity suggests the need to clarify the boundary conditions under which offloading supports versus undermines memory performance. This finding aligns with the transactive memory theory (Sparrow et al., 2011; Wegner, 1987), which suggests that people rely on external systems to store information. Transactive memory systems allow us to share information with people, technology, and objects, forming what has been described as a prosthetic memory. Since participants assumed they would later access the photographs, they relied on the camera to store the information, engaging less in memory encoding. When access to the photos was denied, their recall performance declined (Soares & Storm, 2018).

Research has shown that cognitive offloading can impact cognitive functioning, sometimes yielding benefits but also carrying potential drawbacks. Prior to deciding to offload information, people tend to perform a cost–benefit analysis of its utility (Gilbert et al., 2023). Among its advantages are enhanced focus on other tasks (Soares & Storm, 2018), a higher probability of recalling the offloaded information, and enhanced prospective memory performance, remembering to execute future tasks (Burnett & Richmond, 2023). Overall, cognitive offloading is recognized as a highly effective and practical strategy (Gilbert et al., 2023). Nonetheless, there are potential drawbacks, particularly a decline in information retrieval when external memory aids (e.g., alarms and Post-it notes) are no longer available. A clear example is the photo-taking impairment effect, which suggests that directly observed objects are better remembered than those captured in photographs, particularly when the photos are inaccessible (Henkel, 2014; Soares & Storm, 2018, 2022).

Cognitive offloading has been shown to significantly impact cognitive processes across several domains. This study aims to expand upon the work of Fellers et al. (2023) by refining and extending their paradigm. The work of Fellers et al. (2023) examined the effects of cognitive offloading on a prospective memory task in a two-phase study conducted over a 2-day interval. During the first phase, participants were provided with information on “dry eye syndrome” and its treatment and were instructed to email the researcher at a predetermined time (7 p.m.) two days later. The independent variable (cognitive offloading) had two levels: one in which participants created a reminder and one in which they did not, relying only on their internal memory. Fifteen minutes after the scheduled time (7 p.m.), all participants received an email with a link requesting information about the treatment of the “dry eye syndrome”, regardless of whether they had completed the prospective task. This allowed researchers to evaluate retrospective memory (note that Fellers et al. used videos as stimulus material). The current study contributes to the growing literature on how the timing and nature of offloading influence both prospective and retrospective memory. In particular, it examines whether fully automated forms of offloading (e.g., pre-scheduled actions) differ cognitively from semi-automatic ones (e.g., reminders requiring later initiation). It is important to note that these two forms of offloading are not equivalent: in the reminder condition, only the temporal monitoring component is delegated, while the participant must still retrieve and execute the intended action at the appropriate time. In the scheduled email condition, both the action and its timing are fully delegated immediately, such that no memory component needs to be actively maintained after scheduling. This asymmetry is acknowledged throughout and discussed in terms of its implications for interpreting prospective memory performance. The primary aim of this study was to investigate the impact of reminders on both prospective and retrospective memory. Fellers et al.’s (2023) study showed that reminders increased participants’ probability of emailing the researcher on time and their adherence to the task, compared with participants in the internal condition. Unfortunately, they did not find evidence of the effect of setting a reminder on the retrospective memory task. This result might be explained by the fact that, despite receiving the instructions to set a reminder, they delayed setting it (e.g., an alarm) until after they had finished watching the videos. This can be seen as a critical distinction because the intention to set a reminder might have been sufficient to free up enough cognitive resources for the retrospective memory task.

Although the study by Fellers et al. (2023) provides the basis for the present work, we implemented several key modifications to their original paradigm. Unlike Fellers’ Experiment 2, in which participants received instructions only before watching the video, our study requires participants to receive instructions and set a strategy to see whether this increases performance in the retrospective memory task. We also altered the information presented in the video, using a CBS News video report as the to-be-remembered stimulus. Furthermore, we have expanded the cognitive offloading strategy variable to three levels, the new one being an automatic email scheduling mechanism. This new level was introduced so participants could fully offload the intention, as scheduling the email could remove the need to retain it in memory, allowing them to focus solely on the video, unlike the reminder condition, which still demands some intention maintenance.

This study pursues two main objectives. First, to deepen the research on the impact of cognitive offloading on prospective and retrospective memory tasks, this study builds upon the work of Fellers et al. (2023). Second, to examine the effects of different offloading strategies used on prospective and retrospective memory performance. This leads to our research question, “What is the effect of cognitive offloading on a prospective memory task, considering the strategy implemented?” From this question, we propose four hypotheses: (1) Participants in the reminder and scheduled email condition will have a better performance in the prospective memory task, compared to the participants in the no-reminder condition; (2) Participants who schedule an email (scheduled email condition) will perform better in the retrospective memory task; (3) Participants in the no-reminder condition will engage in more rehearsals than both participants in the offloading conditions (reminder and scheduled email); (4) Participants in both offloading conditions will have higher metacognition than those in the no-reminder condition.

## 2. Materials and Methods

### 2.1. Participants

The sample size was calculated using G*Power 3 (Heinrich Heine University Düsseldorf, Düsseldorf, Germany; Faul et al., 2007) for a One-way ANOVA with three conditions [f = 0.40, α = 0.05, (1 − β) = 0.95] (Faul et al., 2007). This analysis involved a sample of 102 participants, with 34 per condition. However, 152 participants (reminder = 50; scheduled email = 51; no-reminder = 51) were included to compensate for potential non-adherence (i.e., failure to complete the study’s second phase). The participants were recruited through an online recruitment system; therefore, they were university students from the University of Minho (Braga, Portugal), all of whom were adults aged 18–53 years (M = 22.00; SD = 6.11).

All participants provided informed consent before participation. The study protocol was approved by the Ethics Committee of the School of Psychology at the University of Minho (reference CEISCH-135/2024). Eligibility criteria required participants to be fluent in Portuguese, aged 18 years or older, and to report normal or corrected-to-normal vision and hearing. Exclusion criteria included a self-reported history of neurological or psychiatric conditions. Participants received partial course credit as compensation for their participation in the study.

**Figure 1:**
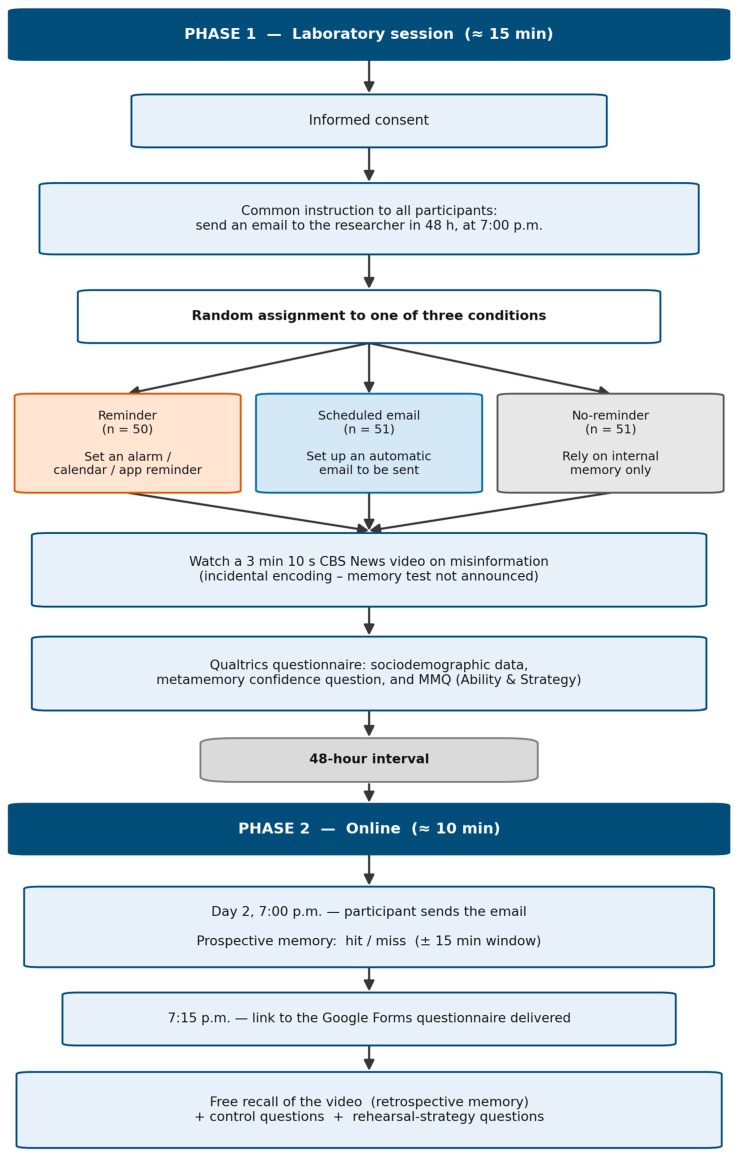
Schematic overview of the two-phase study procedure. Phase 1 was conducted in the laboratory; Phase 2 was completed online 48 h later. MMQ = Multifactorial Memory Questionnaire.

This study employed a two-phase procedure conducted over a 48-h interval to examine the effects of cognitive offloading on both prospective and retrospective memory. In the first phase, held in a laboratory setting, participants were randomly assigned to one of three conditions (reminder, scheduled email, or no-reminder) and instructed to send an email to the researcher 48 h later at 7 p.m. They also watched a short news video about misinformation, which served as the to-be-remembered material for the retrospective memory task. In the second phase, conducted online, the prospective memory task was operationalised by whether participants sent the email within the designated time window. Fifteen minutes after the target time (7:15 p.m.), all participants received a link to an online questionnaire containing a free recall question about the video content (retrospective memory task), along with questions about rehearsal strategies and a metamemory confidence rating. This two-phase design allows for the simultaneous assessment of participants’ ability to remember to perform a future action (prospective memory) and their retention of incidentally encoded information (retrospective memory) as a function of the offloading strategy employed. An overview of the two-phase procedure is presented in Figure 1.

### 2.2. Design

Cognitive offloading was the independent variable, manipulated in a between-subjects design across three conditions: reminder, scheduled email, and no-reminder.

The dependent variables were: (1) performance on the prospective memory task (how accurately the participants remember to send the email); (2) performance on the retrospective memory task (participant’s accuracy in recalling the learned information); (3) rehearsals made during the interval between phases; (4) metamemory, which refers to the confidence participants express when recalling the need to send the email; and (5) adherence (whether the participants completed the retrospective task). Prospective memory performance was operationalized by sending the email within a 15 min window around the target time (7 p.m.). Retrospective memory was measured as the total number of correctly recalled information units from the video. Rehearsals were quantified by participants’ self-reported frequency over the two-day interval. Metamemory was measured as participants’ numerical confidence ratings (0–100), and adherence was defined as completion of the second-phase questionnaire.

### 2.3. Materials

Following the procedure outlined by Fellers et al. (2023), we ensured that the instructions were clear, structured, and consistent throughout the experiment by using a PowerPoint (Microsoft Corporation, Redmond, WA, USA) presentation as a visual aid, highlighting key points of the instructions across the slides.

A CBS (CBS Broadcasting Inc., New York, NY, USA) News report video titled ‘Tracking how dangerous misinformation spreads online’ was shown to participants for later retrospective memory evaluation. This news report explained what misinformation is, provided examples, and offered strategies to stop it from spreading. This topic was chosen due to its heightened relevance in current societal contexts. The video was presented with Portuguese subtitles to ensure full accessibility for all participants. The video duration was 3 min and 10 s. Given its duration, the video was rich in information and detail. It contained 171 possible information units. Information units included both factual content narrated in the video (e.g., definitions, statistics, named examples) and salient visual and contextual features (e.g., the presenter’s appearance, studio setting, and on-screen text). Intrusions were defined as details recalled by participants that were not present in the video (i.e., false recalls or inferences); omissions (i.e., video content not reported) were not counted as intrusions. Each unit corresponded to 1 point; therefore, participants were awarded 1 point for each unit correctly recalled. The final score had the total number of correct information units and intrusions recalled for further analysis. For instance, the response “Studies about the non-existence of climate change, sunscreen and its cancerous effects” received 5 points, corresponding to 5 correct information units.

Another example is “I remember that the video begins with a journalist in a television studio speaking about fake news. I think it was CBS, and there were white footnotes with black letters supporting the news. Then the broadcast cuts to a man who wasn’t in a studio speaking about the topic. I think he had greyish hair and was wearing a dark, long-sleeved shirt. I don’t remember what he said, I only remember that he repeated almost the same sentence at the beginning and at the end of his speech. The broadcast then returns to the studio.”, which was awarded 11 points for correct information units and 2 intrusions (“wasn’t in a studio”; “was wearing a dark long-sleeved shirt”).

Two independent raters, blind to experimental condition, coded the recall data for correct information units and intrusions. Inter-rater reliability was assessed on 25% of the responses (Cohen’s κ = 0.88), indicating excellent agreement. Discrepancies were resolved through discussion. This procedure ensured consistency in recall scoring.

A Qualtrics (Provo, UT, USA) form was organized into sections for collecting sociodemographic data, a metamemory question, and a metamemory scale. The sociodemographic data were assessed by asking participants about their age, sex, nationality, and whether they had vision or auditory problems. Regarding the metamemory question, the participants were given a question regarding the reminder and no-reminder conditions:

*“How confident/sure are you that you will send the email in 2 days, at 7 p.m.? We ask you to tell us a number from 0 to 100. “0” corresponds to I don’t have any confidence/sureness that I will remember to send the email, and “100” corresponds to I have a huge confidence/sureness that I will remember to send the email.”.*

The question that was asked of the participants in the scheduled email condition was slightly different, this one being:

*“How confident/sure are you that the email you programmed will be sent in 2 days, at 7 p.m.? We ask you to tell us a number from 0 to 100. “0” corresponds to I don’t have any confidence/sureness that the email will be sent, and “100” corresponds to I have a huge confidence/sureness that the email will be sent.”.*

To assess metamemory, we utilized a Portuguese version of the Multifactorial Memory Questionnaire (MMQ; Troyer & Rich, 2002; translation by Rooij, 2024). This questionnaire measures memory functioning, self-appraisal of memory ability, and self-reported use of memory strategies using three scales. The items are rated on a 5-point Likert scale based on participants’ experiences over the previous 2 weeks. This study involved only the MMQ-Ability and MMQ-Strategy scales. The MMQ-Ability scale (memory losses) comprises 20 items depicting common memory mistakes. This scale assesses an individual’s self-perception of everyday memory and is scored 0–80 (higher scores indicate better self-reported memory ability). The MMQ-Strategy scale (memory strategy use) comprises 19 items that assess the use of various memory strategies over the previous two weeks. This scale measures practical memory strategies and tools in everyday life. Scores range from 0 to 76 (higher scores indicate greater use of memory strategies). Internal consistency for the Portuguese adaptation of the MMQ was high for both subscales (MMQ-Ability α = 0.89; MMQ-Strategy α = 0.86) (Rooij, 2024). The MMQ-Satisfaction subscale was excluded because it assesses affective responses to memory rather than cognitive performance and was therefore not relevant to the present hypotheses.

Google Forms (Google LLC, Mountain View, CA, USA) was also implemented, in which participants responded to a free recall question asking them to recall as much information as possible about the video. The questionnaire also had some control questions: if they imagined that they were going to be asked to remember information about the video, if they searched for the video on YouTube, if they talked to anybody about the video or the experiment itself, and if they used any reminder besides the one they were told to implement. Lastly, the questionnaire had an explanation about what rehearsals are and some questions to which participants had to respond: if they could give some examples of rehearsals that they had experienced in the last hour; to estimate how many rehearsals about the study they had experienced during the previous two days; and, finally, to give an example about a rehearsal that may have happened that was related to this experiment.

### 2.4. Procedure

This study consisted of two phases separated by a 2-day interval, as per the retention interval proposed by Fellers et al. (2023). The first phase was conducted in the Human Cognition Laboratory at the University of Minho and lasted 15 min. The second phase was conducted online and lasted 10 min. All sessions were conducted individually under standardized conditions, maintaining consistent lighting, noise levels, and screen distance. The experimenter followed a fixed script to minimize interpersonal variability. Participants were instructed not to take notes or discuss the task between sessions to reduce contamination effects.

In the first phase, participants were instructed to sit approximately 50 cm from a computer. First, they had to sign the informed consent form, which contained all the information about the study. After that, they could proceed with the experiment. Initially, all participants received the same instructions: they were told to send an email by 7 p.m. on day 2. Following this, they were randomly assigned to one of three conditions: a reminder, a scheduled email, or no-reminder. In the reminder condition, participants were instructed to set a reminder (e.g., an alarm, a calendar, a reminder app) to send an email 2 days from now at 7 p.m. In the scheduled email condition, they were guided through setting up an automatic email to be sent at the specified time. In the reminder and scheduled email conditions, both the reminders and the automatic email were set at that moment before watching the video. Lastly, in the no-reminder condition, they were instructed not to use external aids to remind them to send the email; therefore, they could rely only on their internal memory to recall the task.

The second part of this phase involved a video presentation. After receiving the instructions, which were supported by a PowerPoint presentation, all participants completed the same initial task. They watched an informative video about misinformation or fake news without being given instructions on how to retain its content for future reference. Importantly, participants were not informed in advance that they would be tested on the video content; the retrospective memory test was therefore incidental and unexpected. Afterward, they had to complete a Qualtrics questionnaire with sociodemographic data, a metamemory question, and a metamemory scale, assessed using two scales of the Multifactorial Memory Questionnaire (MMQ-Ability and MMQ-Strategy). Two days after the first phase, participants would send an email at 7 p.m., and at 7:15 p.m., they would receive an email with a link to an online questionnaire (Google Forms). The questionnaire was composed of (1) free recall instruction, in which the participants were requested to remember and describe the details of the video watched in the first phase of the study; (2) control questions; and (3) the use of rehearsal strategies.

## 3. Results

Statistical analyses were performed using Jamovi 2.6 (The Jamovi Project, 2025, Sydney, Australia). All statistical assumptions were verified prior to analysis. Normality was assessed using Shapiro–Wilk tests, while homogeneity of variances was examined with Levene’s test. When assumptions were violated, non-parametric tests (e.g., Spearman correlations) or Welch’s ANOVAs were used. For categorical variables, chi-square tests of independence were used. Pairwise comparisons were conducted to further analyze differences between conditions. Effect sizes are reported as partial eta squared (η^2^_p_) or Cramer’s V, odds ratio (OR), or Spearman’s rho (r_s_), and 95% confidence intervals are presented where applicable.

### 3.1. Prospective Memory: On-Time Initiation

To assess whether the condition (reminder vs. scheduled email vs. no-reminder) affected the participant’s performance in the prospective task execution (emailing the researcher at 7 p.m., 2 days after the first phase), a chi-square test of independence was conducted. All the participants who responded within the 15 min window, before and after 7 p.m., were considered “hits”, while participants who responded outside that temporal window were considered “misses”. Descriptive analyses showed apparent differences across conditions in on-time initiation rates. The reminder and scheduled email conditions demonstrated substantially higher completion rates than the no-reminder condition (see Table 1). The chi-square test suggests a significant association between the condition and on-time initiation, χ^2^(2, N = 152) = 43.40, *p* < 0.001, V = 0.535.

To further examine these differences, pairwise comparisons were made. Participants in the reminder condition showed a higher proportion of on-time responses (0.84) than those in the no-reminder condition (0.37), χ^2^(1, N = 101) = 23.1, *p* < 0.001, V = 0.478, OR = 0.11, 95% CI [0.04, 0.3]. Similarly, participants in the scheduled email condition (0.92) also outperformed those in the no-reminder condition (0.37) on the prospective task, χ^2^(1, N = 102) = 33.7, *p* < 0.001, V = 0.574, OR = 0.05, 95% CI [0.02, 0.2]. On the other hand, participants had a slightly higher level of performance in the scheduled email condition (0.92) than in the reminder condition (0.84). Still, this difference was not significantly different, χ^2^(1, N = 101) = 1.60, *p* = 0.205, V = 0.126, OR = 2.24, 95% CI [0.63, 8.0]. Overall, both offloading conditions significantly increased the likelihood of performing the prospective task on time compared with relying solely on internal memory. However, no reliable difference emerged between the two offloading strategies, suggesting comparable effectiveness. These results indicate that participants who relied only on their memory were less likely to execute the prospective task on time than those in both offloading conditions. A small subset of participants in the scheduled email condition (N = 4) did not send their emails on time, possibly due to scheduling or system errors. These cases were retained in the analysis as “misses,” in accordance with the intent-to-treat principle.

### 3.2. Adherence

A chi-square test of independence was also performed to evaluate whether the conditions affected adherence and whether participants completed the study. The analysis revealed no significant differences between conditions, χ^2^(2, N = 152) = 0.15, *p* = 0.928, V = 0.031. Therefore, the adherence rates were quite similar between the reminder (0.88), scheduled email (0.90), and no-reminder (0.88) conditions. These results suggest that, even though the implemented condition influenced on-time responses, it did not affect whether participants completed the second task (the retrospective task). This may be explained by the reminder participants received, which instructed them (again) to complete the questionnaire (the second task) to receive course credits, likely motivating them to respond. This may account for the non-significant differences in adherence. Thus, although offloading improved prospective performance, it did not increase overall compliance with the study’s second phase.

### 3.3. Retrospective Memory

Retrospective memory performance was evaluated through a free report question to which participants had to respond. We examined whether the participants’ retrospective memory performance was affected by the condition to which they were enrolled. Given the violation of normality, a one-way Welch ANOVA was performed to compare the amount of correct information units recalled across all conditions. The results showed no significant differences between conditions, F(2, 84) = 1.12, *p* = 0.331, η^2^_p_ = 0.014. Post hoc tests revealed no significant pairwise differences. These results suggest that the type of cognitive offloading (or its absence) did not significantly affect participants’ retrospective memory performance, as shown in the Fellers et al. (2023) study.

We also analyzed the number of intrusions (i.e., details recalled by participants that were not present in the video; omissions were not counted as intrusions). Once again, due to the violation of normality, a one-way Welch ANOVA was conducted. This analysis showed no significant differences between conditions, F(2, 85.2) = 0.065, *p* = 0.937, η^2^_p_ = 0.001. Revealing a similar mean number of intrusions per participant across all conditions: reminder (M = 0.77, SD = 1.03), scheduled email (M = 0.69, SD = 0.97), and no-reminder (M = 0.76, SD = 1.05). Therefore, these results suggest that the condition did not influence the occurrence of memory intrusions in the retrospective memory task.

A one-way Welch ANOVA was performed to compare the three conditions and to examine participants’ precision, defined as the proportion of correct information units divided by the total amount of information recalled. This analysis showed no significant difference between conditions, F(2, 81.8) = 0.428, *p* = 0.653, η^2^_p_ = 0.009. The results also reveal similar performance across all conditions: reminder (0.94), scheduled email (0.91), and no-reminder (0.93). This suggests that the condition did not affect participants’ retrospective recall accuracy.

These complementary analyses (correct information, intrusions, and precision) were conducted to capture both the quantity and quality of recall. Together, they suggest that the manipulation did not affect either the accuracy or the completeness of retrospective memory. These results replicate previous findings (Fellers et al., 2023), reinforcing that cognitive offloading enhances prospective but not retrospective recall.

### 3.4. Rehearsals

Before this analysis, 10 outliers were removed to prevent extreme values (e.g., 100 rehearsals). Outliers were defined as values exceeding three standard deviations from the group mean on reported rehearsals. Eliminating these cases reduced skewness and improved the distribution’s normality without altering the overall pattern of results.

Due to violations of normality, a one-way Welch ANOVA was performed to assess whether the number of reported rehearsals differed significantly between conditions. The results reported significant differences between conditions, F(2, 74.0) = 8.32, *p* < 0.001, η^2^_p_ = 0.137. Games-Howell post hoc analysis indicated that the no-reminder condition (M = 5.77, SD = 5.39) had significantly more rehearsals than the reminder (M = 3.50, SD = 1.78) and scheduled email (M = 2.57, SD = 1.64) conditions. These results suggest that participants in the no-reminder condition, who relied solely on their memory, made more rehearsals than those in both offloading conditions. This finding supports the hypothesis that participants without external aids compensate by engaging in more frequent internal rehearsal.

### 3.5. Metamemory

To assess participants’ confidence in sending the email, or in having the email sent, we performed a one-way Welch ANOVA due to violations of the assumption of normality. This analysis revealed a significant difference between conditions, F(2, 92.4) = 25.4, *p* < 0.001, η^2^_p_ = 0.341. Post hoc comparisons showed that participants in both the reminder (M = 88.9, SD = 11.4) and scheduled email (M = 92.7, SD = 10.3) conditions rated their memory confidence significantly higher than those in the no-reminder condition (M = 64.5, SD = 26.2). No significant differences were observed between the reminder and scheduled email conditions (*p* = 0.187). These findings suggest that external aids (e.g., alarms, reminder apps, calendars) may enhance confidence in memory performance. Overall, the presence of external aids increased participants’ confidence in their ability to complete the prospective task.

In summary, cognitive offloading significantly improved prospective task performance and subjective confidence but did not influence retrospective recall or overall adherence. Participants without external aids compensated through more frequent rehearsals, which supports the metacognitive regulation account of offloading behavior.

### 3.6. Correlations Between Cognitive Measures

Spearman correlation analyses were conducted to explore the possible relationships between self-reported metamemory and both scales of the MMQ (e.g., ability and strategy scales), as the self-reported metamemory variable did not follow a normal distribution across all conditions. Participants who reported higher confidence in remembering the prospective task tended to score higher on the MMQ—Ability scale, r_s_ = 0.273, *p* < 0.001, but, on the other hand, the reported confidence variable did not seem to relate to the use of memory strategies (MMQ—Strategy scale), r_s_ = 0.056, *p* = 0.494. According to the data, both MMQ scales were significantly and negatively correlated (r_s_ = −0.467, *p* < 0.001). This result might suggest that participants who reported a higher “everyday memory ability” tended not to rely as much on memory strategies.

In terms of prospective task performance, Spearman correlations revealed a significant positive correlation between on-time initiation and self-reported metamemory (confidence in remembering to send the email; r_s_ = 0.436, *p* < 0.001). This result suggests that individuals who rated themselves as having higher confidence in their memory to send the email tended to perform better on the prospective memory task (sending the email on time). On the other hand, adherence to the second part of this study was not significantly correlated to any of the measured variables, for example, on-time initiation (r_s_ = 0.142, *p* = 0.082).

Regarding retrospective performance, the number of correctly recalled information units from the previously watched video was not correlated with either self-reported metamemory or both MMQ scale scores. Nevertheless, participants with more frequent use of memory strategies (higher MMQ-Strategy scores) tended to produce more intrusions (r_s_ = 0.238, *p* = 0.005), and there was a tendency for participants with higher memory ability to produce fewer intrusions (r_s_ = −0.178, *p* = 0.039).

Together, these results suggest that perceived memory ability (MMQ–Ability) aligns with greater confidence and actual prospective performance, while the use of compensatory strategies (MMQ–Strategy) may be inversely related to self-perceived ability.

## 4. Discussion

This study examined the impact of cognitive offloading on prospective and retrospective memory, extending earlier paradigms (Fellers et al., 2023) by introducing a misinformation domain and a fully automated offloading condition. Participants were randomly assigned to one of three conditions (reminder, scheduled email, or no-reminder) and instructed to send an email 48 h after an initial meeting (prospective memory task). At that point, they also completed a free recall questionnaire about a previously watched video on misinformation detection (retrospective memory task). Following Fellers et al. (2023), this design allows us to examine the relationships among cognitive offloading strategies, rehearsal, metamemory, and their influence on both memory components.

As expected, participants who offloaded were more likely to email the researcher at the designated time, producing results similar to those of Fellers et al. (2023) on prospective memory performance. Therefore, participants in both offloading conditions (reminder and scheduled email) demonstrated higher performance in the prospective memory task (emailing the researcher on time) than participants who relied solely on their memory (no reminder). These results show the efficacy of completing a prospective task when setting a reminder in a naturalistic context, thus confirming our first hypothesis, which suggests that the use of external reminders reduces cognitive load and enhances the likelihood of pursuing a future intention (Burnett & Richmond, 2023; Soares & Storm, 2018).

There were no significant differences in adherence (completing the second phase of the study, the retrospective task) across conditions; most participants completed the task in all conditions. We obtained contrasting adherence results compared with those of Fellers et al. (2023). These results can be explained by the researcher’s second reminder to complete the second task to receive course credits (only participants who completed the retrospective task received course credits), which may have attenuated group differences. This methodological aspect (second reminder) differs from Fellers et al. (2023), who did not send a second reminder for the retrospective task. For future studies, it would be important to avoid sending a second reminder to determine whether this influences adherence and to observe any differences between the three conditions. According to the social perspective that Fellers et al. (2023) follow (e.g., cognitive dissonance: Harmon-Jones & Harmon-Jones, 2007; saying-is-believing theory: Higgins & Rholes, 1978), the reminder condition would have a higher adherence in completing the retrospective task, since setting a reminder might increase the perceived importance and could influence their subsequent behavior (completing the retrospective task). From this perspective, we believe that the reminder condition would lead to higher adherence to the second task than the scheduled email and no-reminder conditions, as there may be a drive to remain consistent and successful across both tasks, thereby complying with the second task to complete the retrospective task.

Contrary to our second hypothesis, neither cognitive offloading condition resulted in better performance on the retrospective memory task than the no-reminder condition. This result is consistent with previous findings, such as those of Fellers et al. (2023) and Kvavilashvili (1987), which indicate that retrospective memory performance is independent of prospective memory performance. Since participants in the scheduled email would fully offload their intention, not needing to retain it actively, we believe this would enable them to focus entirely on the information transmitted in the video, thereby improving their retrospective memory performance. Contrary to expectations, no differences were observed between the reminder and scheduled email conditions. One possible explanation for these statistically nonsignificant results is familiarity with the information in the video (how to identify and prevent the spread of misinformation), since misinformation is a widely discussed topic (Allcott & Gentzkow, 2017; Lazer et al., 2018). Furthermore, because participants were not informed in advance that their memory for the video would be tested, encoding was incidental; any cognitive capacity freed by offloading could have been allocated to activities other than deep encoding (e.g., mind wandering, planning other tasks), which may partly account for the null retrospective memory results. Rather than recalling the information in the video, participants may have recalled prior knowledge about the issue. Our results are similar to those of Fellers et al. (2023), as no statistically significant differences were found in either study. Whilst Fellers et al. (2023) reported similar means across both conditions (Offloading: M = 0.43; Internal: M = 0.43), our study also didn’t find significant differences in retrospective memory performance between conditions (Reminder: M = 10.66; Scheduled email: M = 8.44; No reminder: M = 9.91). These null findings align with the idea that offloading can impair internal encoding when individuals assume external storage will be available (Henkel, 2014; Sparrow et al., 2011). Participants in both offloading conditions may have relied more on the external system, leading to shallower encoding of the video content. Thus, while offloading supports the execution of intentions, it may simultaneously weaken the episodic encoding of unrelated information. To further research these results, future studies may investigate whether resorting to more unfamiliar information and detail-specific stimuli can yield significant differences in recall accuracy across cognitive offloading strategies. This pattern supports the notion that offloading functions as a cognitive load management strategy, allowing attentional and working memory resources to be redistributed toward ongoing tasks. Consistent with resource theories of prospective remembering (Einstein & McDaniel, 2005), external reminders likely reduced the need for time-based monitoring, facilitating more spontaneous retrieval when the cue appeared. This dissociation may also reflect, at least in part, the distinct neural systems that support each form of memory. Prospective memory, particularly the formation and maintenance of delayed intentions, has been consistently associated with the rostral prefrontal cortex, approximating Brodmann Area 10 (Burgess et al., 2011; Simons et al., 2006), whereas the encoding and retrieval of episodic, retrospective content rely more heavily on medial temporal lobe structures, including the hippocampus (Cabeza & Nyberg, 2000; Squire, 1992). Because cognitive offloading primarily reduces the strategic monitoring and self-initiated retrieval demands supported by prefrontal regions, it would not be expected to enhance the medial temporal lobe processes that underlie retrospective recall. This partial neural dissociation offers a plausible account of why prospective memory offloading did not translate into improved retrospective memory performance in the present study.

Confirming our third hypothesis, individuals who relied solely on their memory engaged in more nonintentional reminders of a future intention, or rehearsals, than those who used external tools. This result supports the idea that when we cannot rely on external tools, we tend to compensate for potential lapses in memory by engaging in cognitive strategies to monitor and sustain our intentions, in this case, through rehearsals (Fellers et al., 2023; Zogg et al., 2012). This compensatory use of rehearsal is consistent with metacognitive control models (Koriat & Goldsmith, 1996), which posit that individuals monitor their perceived memory reliability and deploy additional internal strategies when confidence is low or external support is absent.

Lastly, we confirmed our fourth hypothesis; therefore, both offloading conditions had higher levels of confidence in sending the email on time or that the email would be sent (scheduled email condition) when compared to the no-reminder condition, which supports the idea that external aids might increase confidence in prospective memory performance (Gilbert et al., 2023). Importantly, it should be noted that for the scheduled email condition, confidence ratings reflect trust in the technology (that the automated system would function correctly) rather than metacognitive monitoring of one’s own memory. These two forms of confidence should therefore not be treated as equivalent. The correlation between metamemory and prospective memory performance indicated a relationship between the two, suggesting that individuals with higher confidence in remembering to send the email tended to perform better on the prospective memory task. These findings raise important questions about metacognitive calibration: although confidence was higher in offloading conditions, it is unclear whether participants’ confidence accurately tracked actual performance. Prior work (Boldt & Gilbert, 2019) suggests that external aids can create an “illusion of competence,” leading individuals to overestimate the reliability of their memory when supported by technology. Future research should investigate whether such overconfidence impacts real-world task management.

Taken together, the findings of this experiment suggest a double-edged role of cognitive offloading: while external aids reliably enhance prospective remembering and subjective confidence, they do not necessarily strengthen, and may even undermine, the encoding of concurrent or contextual information. They can also offer important insights that can be translated into practical implications. In applied contexts, these findings underscore the need to design reminder systems that not only support the execution of intentions but also promote the deeper encoding of related information. For instance, labeling digital reminders with contextual cues (e.g., “take medication after breakfast”) may enhance both prospective and retrospective memory components. Although most findings indicate a positive relationship between cognitive offloading and prospective memory, specific strategies within cognitive offloading, such as reminders with descriptive labels, may enhance retrospective memory by presenting context-specific cues at retrieval (Morrison & Richmond, 2020; Richmond et al., 2023). Therefore, cognitive offloading may benefit both prospective and retrospective memory; however, further studies are needed to understand its impact on the retrospective component to avoid potential memory lapses.

Future research should systematically manipulate the familiarity and emotional salience of to-be-remembered materials to test whether cognitive offloading benefits depend on encoding depth. Moreover, expanding samples beyond university students could reveal developmental or individual-difference effects, such as how age, technological reliance, or metacognitive ability modulate offloading efficacy. In addition, future studies could enhance ecological validity by allowing participants to select their own offloading strategy rather than being assigned to a fixed condition. This approach would make it possible to examine how many participants spontaneously choose to fully offload an intention (e.g., the scheduled email) and whether this choice aligns with their metamemory beliefs. Similarly, incorporating self-generated or mixed prospective memory tasks, although reducing experimental control, would more closely reflect the variety of intentions that people manage in everyday life.

Overall, the present findings contribute to a growing body of evidence that cognitive offloading enhances the prospective component of memory, while leaving the retrospective component largely unaffected. This dissociation highlights the importance of distinguishing between remembering when to act and remembering what to act upon, two processes that may rely on distinct cognitive and metacognitive mechanisms. Although further research is needed, the present findings highlight the importance of cognitive offloading for the behavioral sciences and open avenues for future studies by suggesting experimental procedures to better understand when and how offloading optimally supports memory performance.

## Figures and Tables

**Table 1:** Results by Condition.

Measure	Scale/Range	Reminder	Scheduled Email	No-Reminder	Test Statistic; *p*-Value
On-time initiation (Hits)	% of participants	84%	92%	37%	χ^2^ = 43.4; *p* < 0.001
Adherence	% of participants	88%	90%	88%	χ^2^ = 0.149; *p* = 0.928
Confidence (On-time initiation)	Confidence rating, 0–100	88.9 (11.4)	92.7 (10.3)	64.5 (26.2)	F(2, 92.4) = 25.4; *p* < 0.001
Rehearsals	Frequency count, M (SD)	3.50 (1.78)	2.57 (1.64)	5.77 (5.39)	F(2, 74.0) = 8.32; *p* < 0.001
Retrospective Memory	Correct units, M (SD)	10.66 (8.84)	8.44 (5.77)	9.91 (8.70)	F(2, 84.0) = 1.12; *p* = 0.331

Note. Values for on-time initiation and adherence are percentages of participants per condition; values for confidence, rehearsals, and retrospective memory are means with standard deviations in parentheses. The Scale/Range column indicates the metric of each measure: on-time initiation and adherence are dichotomous (hit/miss and completed/not completed, respectively); confidence is a self-reported metamemory rating ranging from 0 (no confidence) to 100 (complete confidence); rehearsals is the self-reported frequency of spontaneous task-related thoughts over the 48 h interval; and retrospective memory is the number of correctly recalled information units, out of 171 possible units. Test statistics are chi-square tests of independence for the dichotomous measures and Welch’s one-way ANOVAs for the continuous measures; n = 50 (reminder), 51 (scheduled email), and 51 (no-reminder).

## Data Availability

The de-identified quantitative dataset supporting the findings of this study (condition assignment, prospective-memory outcomes, adherence, coded retrospective-memory scores, rehearsal counts, metamemory confidence ratings, and MMQ-Ability and MMQ-Strategy scores) is openly available in the Open Science Framework (OSF) repository at [repository link to be inserted upon acceptance]. The raw open-ended recall responses are not publicly available, because free-text recall may contain idiosyncratic or potentially identifying information; these responses are available from the corresponding author (P.B.A.) upon reasonable request. The data of this study is available at the following OSF link: https://osf.io/7d5x8/overview?view_only=2dae7d4a1c454336b8e9f936e2604b6d (accessed on 1 March 2025).
